# Using Unstated Cases to Correct for COVID-19 Pandemic Outbreak and Its Impact on Easing the Intervention for Qatar

**DOI:** 10.3390/biology10060463

**Published:** 2021-05-24

**Authors:** Narjiss Sallahi, Heesoo Park, Fedwa El Mellouhi, Mustapha Rachdi, Idir Ouassou, Samir Belhaouari, Abdelilah Arredouani, Halima Bensmail

**Affiliations:** 1 National Institute of Posts and Telecommunications (INPT), Rabat 210024, Morocco; narjiss.sallahi@gmail.com; 2 Qatar Environment and Energy Research Institute, Hamad Bin Khalifa University, Doha P.O. Box 34110, Qatar; hpark@hbku.edu.qa (H.P.); felmellouhi@hbku.edu.qa (F.E.M.); 3 Data Sciences Project, University of Grenoble, 38400 Grenoble, France; mustapha.rachdi@univ-grenoble-alpes.fr; 4 University Qaddi Ayyad, Marrakech 40000, Morocco; i.ouassou@uca.ac.ma; 5 ICT Department, Hamad Bin Khalifa University, Doha P.O. Box 34110, Qatar; sbelhaouari@hbku.edu.qa; 6 Diabetes Department, Qatar Biomedical Research Institute, Hamad Bin Khalifa University, Doha P.O. Box 5825, Qatar; aarredouani@hbku.edu.qa; 7 Data Analytics Department, Qatar Computing Research Institute, Hamad Bin Khalifa University, Doha P.O. Box 5825, Qatar

**Keywords:** coronavirus, COVID-19, reproduction number, transmission rate, reported and unreported cases, interventions, SIR model

## Abstract

**Simple Summary:**

A modified SIR model was applied to provide COVID-19 pandemic analysis and predictions for Gulf Cooperation Council countries, as well as representative countries in Europe and New York City. We estimated reported, infected, and unreported cases from cumulative reported cases and simulated data. We also estimated the basic reproduction rates at different phases of the pandemic. Outputs show that the modified SIR model fits very well with the outcome of the COVID-19 pandemic for the studied countries and could be generalized to other countries. The model prediction emphasizes the value of significant interventions in public health in regulating the epidemic taking into account that a constant fraction of the infected cases remain unreported during the pandemic. We report and analyze the effectiveness of preventive/intervention measures applied to the overall community to curb the severity of the pandemic. Our model could be used to support public health authorities with respect to post-outbreak reopening decisions, highlighting effective measures that need to be maintained, eased, or implemented to support safe reopening strategies in the GCC countries.

**Abstract:**

Epidemiological Modeling supports the evaluation of various disease management activities. The value of epidemiological models lies in their ability to study various scenarios and to provide governments with a priori knowledge of the consequence of disease incursions and the impact of preventive strategies. A prevalent method of modeling the spread of pandemics is to categorize individuals in the population as belonging to one of several distinct compartments, which represents their health status with regard to the pandemic. In this work, a modified SIR epidemic model is proposed and analyzed with respect to the identification of its parameters and initial values based on stated or recorded case data from public health sources to estimate the unreported cases and the effectiveness of public health policies such as social distancing in slowing the spread of the epidemic. The analysis aims to highlight the importance of unreported cases for correcting the underestimated basic reproduction number. In many epidemic outbreaks, the number of reported infections is likely much lower than the actual number of infections which can be calculated from the model’s parameters derived from reported case data. The analysis is applied to the COVID-19 pandemic for several countries in the Gulf region and Europe.

## 1. Introduction

The first known COVID-19 case was reported by officials in Wuhan City, China, on 31 December 2019. Following that date, the number of confirmed infectious cases was on the rise. Consequently, on 11 March 2020, the World Health Organization (WHO) declared the COVID-19 outbreak to be a pandemic [1]. The recorded cases of COVID-19 have increased exponentially worldwide since then, reaching more than 100 million confirmed cases in 191 countries and more than two million deaths globally. As of 23 January 2021, the outbreak has had an effect on 7,119,570 people in the Middle East, and 1,181,199 confirmed cases have been reported in the Gulf Cooperation Council (GCC) countries according to the Worldometers website www.worldometers.info/coronavirus/ (accessed on 1 April 2020). A national emergency was declared for the Coronavirus outbreak in Qatar at the beginning of March 2020 [2], and by 23 January 2021, the number of cases reported in Qatar had already reached 148,772 cases. Following the announcement of COVID-19 as a pandemic, most of the Gulf countries scaled up their responses and put in place rigorous controls [3]; the declaration of a pandemic generally leads to governments spending more on preventive measures and funding for vaccination programs.

Given the gravity of the situation resulting from the COVID-19 pandemic, a fundamental and yet critical question remains unanswered: how many people are currently infected with COVID-19 in the GCC countries and what is the actual value of the basic reproduction number? During the first few months of the outbreak, only a sub-sample of individuals with significant symptoms or travel history could be assessed by hospitals and disease control centers because of the shortage in testing kits; the number of recorded infections is probably much lower than the actual number of infections, particularly early in the course of the pandemic [3,4,5,6]. These unreported infections can, indeed, remain unnoticed, since they often have mild or no symptoms, which can be confused with seasonal flu, for example. If not hospitalized or quarantined, unreported COVID-19 carriers could infect a large proportion of the population, posing genuine difficulties for contact tracing measures and the pandemic’s overall containment. Accordingly, in order to determine the efficacy of preventive measures such as quarantine and social distancing, calculating the number of unreported infections will serve to notify policymakers about the correct scale of virus control policies, school closing, mask-wearing, etc., in slowing down the spread of the epidemic [7,8].

Ideally, a randomized testing experiment would offer an impartial estimation of the infection fatality rate. However, given the limited availability of test kits and the rising demand among people with symptoms, randomized testing, especially in the early periods of the outbreak, may not be feasible. Therefore, it may be of great benefit to estimate and to be able to measure the fraction of unreported infections with observational data on hand. Policymakers will be better prepared to enforce the required degree and duration of virus control policies with this information.

The estimation of results based on model analysis for GCC countries reveals that the basic reproduction number may be as high as 8.9 (95% CI 1.71–8.98) [9]. Sensitivity analysis reveals that the strict controls put in place can effectively decrease the basic reproduction number and the risk of contagiousness, with the impact of travel restrictions on COVID-19 infection in Qatar being almost equal to raising the baseline value of quarantine by 100 thousand baseline value.

As a result, it is critical at this point to accurately assess the reported and unreported infected cases, as this is critical in estimating the basic reproduction number correctly. This would also assist the GCC countries in general, and the Qatari authorities in particular, in evaluating the economic and societal consequences of implementing resource-intensive preventive measures such as the closure of borders, critical businesses, and educational institutions, quarantine, social distancing, the mandatory wearing of protective masks, and upgrading medical tools and equipment. All the above measures have been proven to be efficient for the prevention and the control of the COVID-19 infection, but come with a sizable economic and societal burden, making it essential to continuously evaluate how long these measures should be maintained to lower the infection rate and to prevent the collapse of the country’s health care system.

## 2. Materials and Methods

Typically, reported cases represent a subset of the total number of infected cases, as sometimes only the most severe cases of symptoms are reported. On the basis of Liu et al. (2020) [6,10], our approach is based on the knowledge of the data of new reported cases (typically weekly) over the time course of the pandemic.

The model used here is based on the conventional SIR model, which is an Ordinary Differential Equations (ODE) of the basic SIR model and is a commonly used deterministic model that describes the movement of people through three mutually exclusive phases: *S* (susceptible), *I* (infected) and *R* (recovered).

Classical disease modeling actually uses the continuous differential method, as follows:

(1) $$\{\begin{array}{c} \frac{\partial S\left(t\right)}{\partial t}=-\tau S\left(t\right)I\left(t\right)\\ \;\;\;\frac{\partial I\left(t\right)}{\partial t}=\tau S\left(t\right)I\left(t\right)-\nu I\left(t\right)\\ \frac{\partial R\left(t\right)}{\partial t}=\nu I\left(t\right) \end{array}$$

where the population sizes of *S*, *I*, and *R* individuals change between the times *t* and *t* + *dt*.

We consider the evolution of the virus over a period of time; $t_{0}\leq$ t≤ $t_{1}$. In the early stages of the epidemic, we consider a relatively short period of time. Thus, during this period, the “recovered” population at the epicenter, which is a small fraction of the population, is believed to not play a significant role. The parameter τ>0 corresponds to the disease transmission rate, and the parameter ν>0 corresponds to the removal rate of infected individuals. Typical initial conditions of the model are *S*(0) = $S_{0}$> 0 and *I*(0) = $I_{0}$ > 0 [11].

Initially, the WHO estimated the basic reproduction number for COVID-19, gobally, to be between 1.4 and 2.5. Moreover, several articles aimed to more precisely estimate the value of $R_{0}\;$for COVID-19; see [12,13,14,15,16,17,18,19,20] (Table 1).

### 2.1. Parameters of the Model

Our model uses a compartment for unstated or unreported cases, which is very important when modeling several pandemics. Recent studies have shown that it is important to estimate this number. Saxa et al., 2020 [21] showed that only 2.4% of the cases in India were being reported (i.e., an understatement factor of about 42). Wu et al., 2020 [22] reported that the actual number of infections was estimated to be 3 to 20 times higher than the confirmed cases for various states of the United States of America. Lu et al., 2020 [23] showed that there were drastic cases of underreporting in many countries worldwide.

Therefore, for policymakers to effectively develop strategies for resource distribution, intervention implementation, and promotion of public awareness, accurate predictions of the magnitude and the development of an epidemic are needed.

To develop adequate and valuable modeling, we adapted simple and complementary SIR differential models, which were originally proposed by Bernoulli [24], and adapted by Liu et al., 2020 [6,7]. Most pandemics follow a non-Markovian mechanism, which means that the past states of dynamical systems lead to the current states, which is referred to as “memory” [25]. However, previous knowledge of the prevalence of an outbreak and precautions are not always available or disseminated, and thus people prefer to adopt new approaches to address the disease [26,27,28,29]. Here, instead of using continuous time evolution differential equations, we focused on a time-discrete Markovian process, using the widely applied assumption that days are a natural unit of measurement [30]. This was described using a Markov epidemic process, in which the state of individuals at each time step does not depend on the previous steps. Hence, the model can be described as follows:

$$\{\begin{array}{c} \frac{\partial S\left(t\right)}{\partial t}=-\tau S\left(t\right)\left[I\left(t\right)+U\left(t\right)\right]\\ \;\;\;\frac{\partial I\left(t\right)}{\partial t}=\tau S\left(t\right)\left[I\left(t\right)+U\left(t\right)\right]-\nu I\left(t\right)\\ \frac{\partial R\left(t\right)}{\partial t}=\nu_{1}I\left(t\right)-\eta R\left(t\right)\\ \frac{\partial U\left(t\right)}{\partial t}=\nu_{2}I\left(t\right)-\eta U\left(t\right) \end{array}$$

Based on the reported cases R, this approach recaptures the number of unreported cases U that were not accounted for as a result of factors such as their being asymptomatic or mild cases, unreported contamination, or the mobility of infected individuals (especially at the beginning of the pandemic outbreak) in the GCC. We complemented this with the model of Demongeot et al. [31], which accounts for the birth/death rate since the onset of the pandemic in late winter 2019, and which has continued to surge, with a second wave hitting northern hemisphere countries in fall 2020/winter 2021. The natural death rate was parameterized with μ , and the number of births per unit time was parametrized with Λ. Combining [7] and [31] helped us check whether the number of unreported cases influenced the severity of the pandemic, and to what extent public health measures such as quarantine contribute to containing the pandemic. The modified model can be described as follows:

(2) $$\{\begin{array}{c} \frac{\partial S\left(t\right)}{\partial t}=-\tau S\left(t\right)\left[I\left(t\right)+U\left(t\right)\right]-\mu S+\Lambda \\ \frac{\partial I\left(t\right)}{\partial t}=\tau S\left(t\right)\left[I\left(t\right)+U\left(t\right)\right]-\left(\nu_{1}+\nu_{2}+\mu \right)I\left(t\right)\\ \frac{\partial R\left(t\right)}{\partial t}=\nu_{1}I\left(t\right)-\eta R\left(t\right)\\ \frac{\partial U\left(t\right)}{\partial t}=\nu_{2}I\left(t\right)-\eta U\left(t\right)\\ \frac{\partial N\left(t\right)}{\partial t}=\Lambda -\left(1-\alpha \right)\nu I-\mu N \end{array}$$

where *N = S + I + R + U* [11], with a contact rate of mass action, a constant number of births Λ per unit time, a proportional natural death rate μ in each class, and a rate of recovery or disease death ν of infectives recovering with acquired immunity from reinfection with a fraction α of infectives.

We examine the first four equations in order to determine *S*, *I*, *R*, and *U*, and then consider the fifth equation in order to determine *N* once *S* and *I* are known. This is possible because *N* is not a parameter in the first four equations.

**Figure 1:**
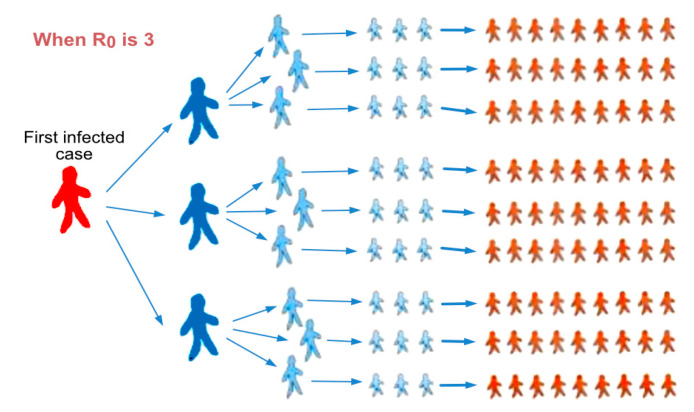
The basic reproduction number (*R*_0_) represents an indication of the initial transmissibility of the virus. Courtesy of Qatar Biomedical Research Institute (QBRI).

The quantity denoted as $R_{0}$ represents the basic reproduction number, also called the basic reproduction ratio or rate (Figure 1) [32]. It is an epidemiological metric used to describe the contagiousness or transmissibility of the virus, i.e., the average number of secondary infections produced by each infected person. It depends on the specific disease (parameter determination ν) and the rate of contacts, as will be described below, depending on the population density in the group being studied [33,34]. The model of illness exhibits a threshold activity: if the basic reproduction number is lower than one, there will be a decline in the number of cases; if $R_{0}$ = 1, the disease is endemic; but if the basic reproduction number is greater than one, the disease will become a pandemic.

Our contribution consists of the development of a modified SIR model that uses reported case data, both asymptomatic and symptomatic, to model the transmission dynamics of the COVID-19 pandemic for some GCC countries, as well as for some representative countries in Europe, along with New York City, taking into consideration the rate of unreported cases and the importance of its estimation in affecting the actual spread of the virus and the level of measures that have been taken to face it. In this context, we estimated the basic reproduction rates $R_{0}\;\mathrm{and}\;R_{e}.\;$The objective of the analysis was to identify the early phases of epidemics, to predict subsequent phases and the shape of their evolution, while incorporating unreported cases into the transmission dynamics. Our contribution also aims to highlight the effectiveness of the implementation of major public policies that restrict social movement with the aim of achieving a time-dependent exponential decrease in the number of cases, supporting safe reopening strategies in the GCC countries.

**Figure 2:**
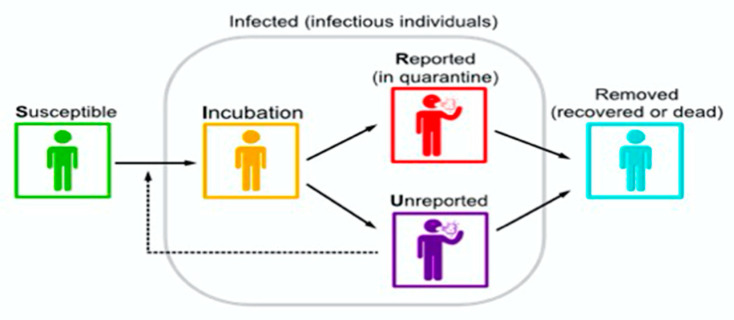
Flow chart of SIR model adding a new sub-compartment related to unreported cases.

The observed data consist of the cumulative reported cases at time *t*, denoted by C(*t*), which corresponds to the total number of reported infectious cases up until time t. We assume that these cumulative cases recorded at time *t* consist of a constant fraction over time of the total number of infected cases up to time *t* in order to handle these data. Furthermore, we assume that the removal rate ν is the sum of the recovery numbers of the reported and unreported cases, following the form $\nu =\nu_{1}+\nu_{2}$, where $\nu_{1}\;$is the removal rate of reported infected individuals and $\nu_{2}$ is the removal rate of infected individuals due to all other causes, such as mortality, recovery or other reasons (Figure 2).

To the best of our knowledge, there have been a limited number of articles published on the prediction and the calculation of the basic reproduction number $R_{0}$ in the context of the COVID-19 pandemic in the Middle East, and particularly in the GCC countries, especially with the inclusion of the unreported cases parameter in SIR. Rahman et al. (2020) [35] used a classic SIR model with least-square error, and reported the *R*_0_ values for Kuwait, Bahrain, Qatar, Saudi Arabia, United Arab Emirates (UAE) and Oman to be 2.71, 3.39, 4.18, 4.45, 2.75, 2.60, respectively. Al-Shammari et al. (2020) [36] used a dynamic transmission SEIR model for Kuwait that was informed by two local mechanisms: a delay period during which suspected COVID-19 individuals are tested, identified, and hospitalized; and different severities of illness. They calibrated the model with a maximum likelihood framework and produced an *R*_0_ ranging between 1.5 and 3.5.

On the other hand, Al Wahaibi et al. (2020) [37] proposed a probabilistic model for modeling dynamic *R*_t_ in Oman. They fitted a Gamma distribution to the susceptible cases and used a Poisson likelihood estimation to capture the transmission of the infection. Their estimated *R*_t_ ranged between 0.9 and 4.65, depending on the non-pharmaceutical intervention period between February and June. Billah et al. (2020) [38] used a meta-analysis model and estimated the *R*_0_ to be 2.87.

Moreover, the GCC population has a combination of unique characteristics when compared to countries from the northern hemisphere, such as high fertility (birth), a young population, and the high prevalence of diseases such as diabetes. Advanced numbers estimation based on model analysis without considering unreported cases for countries in the Middle East reveals that the basic reproduction number may be as high as 8.9 (95% CI 1.71–8.98) (see [39]).

The first stage of the analysis is to note that Model (2) presents a properly posed problem. This is a model in dimensionless time t. It this way, ν becomes a dimensionless parameter. Since *I*(*t*) is integrable on [0, ∞), I(∞) = 0.

That is, since ∂S/∂t ≥ 0 if *S* = 0 and ∂I/∂t ≥ 0 if *I* = 0, we have *S* ≥0, *I* ≥0, for *t* ≥0. Thus, the solution always remains in the biologically realistic region *S* ≥0, I ≥0. Using parameters related to baseline calculation such as $S_{0}$, the number of people susceptible in the population before the epidemic outbreak, which can be approximated here on the basis of the total population size, allows us to obtain accurate information about the values $I_{0}$, τ and ν, as well as the basic reproductive number of the epidemic *R*_0_. We note that these infected-but-unreported individuals are able to spread the virus to susceptible individuals, in contrast to the infected-and-reported individuals, who are isolated in quarantine.

Table 2 summarizes the parameters of the SIR model used in this paper. We modeled the epidemic in several countries in the Gulf region, and added France, Italy and New York for comparison purposes using data from https://www.worldometers.info/coronavirus/Worldometers for Covid-19 (accessed on 1 April 2020).

### 2.2. Fitting the Model to the Data

To fit the model, here we use data from cumulative reported cases to determine the total number of cases *S*(0)–$S_{\infty }$ over the course of the epidemic, as well as the parameters τ,$\;\nu_{1}$, and $\nu_{2}$. The cumulative number of both reported and unreported cases at time *t* is *C*(*t*) =$S_{0}+I_{0}-S\left(t\right)$, and the cumulative number of unreported cases at time *t* is $C_{u}\left(t\right)=C\left(t\right)-C_{R}\left(t\right)$, where $C_{R}\left(t\right)$ is the cumulative number of reported cases. On the other hand, we assume that *C*(*t*) has an exponential behavior *C*(*t*) = $\chi_{1}exp\left(\chi_{2}t\right)+\chi_{3}$.

We fix $S_{0}$, which corresponds to the total population of a given country (for example, for the state of Qatar,$\;S_{0}=2,881,053$). We assume that the value of *S*(*t*) varies insignificantly during the period considered, and we fix ν, η, f, Λ, α and μ. We estimate the parameters $\nu_{1},\;\nu_{2},\;\tau \;$and the initial conditions $U_{0}$ and $I_{0}\;$on the basis of the cumulative reported cases *C*(*t*) (see Appendix A.1 and Appendix A.2). Subsequently, we constructed numerical simulations using Model (2), for comparison with the observed data.

In the following section, we estimate the parameters $\chi_{1}$,$\;\chi_{2}$,$\;\chi_{3}$ and $t_{0}$ using the cumulative reported symptomatic infectious cases. We carefully chose an interval that fits well the exponential curve, as seen in Table 3 for the case of Qatar.

### 2.3. Self Starting Function for the Cumulative Function

**Figure 3:**
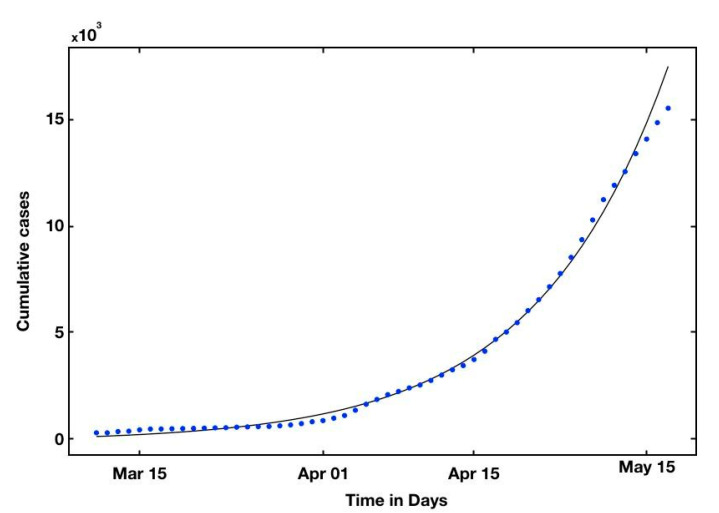
We plot the simulated number of t → C(t) (black line) and the data corresponding to the confirmed cumulative reported cases between 11 March and 16 May (blue dotted) in Qatar. The dots correspond to t → C(t), where C(t) is taken from the cumulated confirmed cases in Table 3 (top).

Applying a log transformation to estimate the three parameters requires a special treatment for $\chi_{3}$. In other words, we cannot convert our cumulative reported exponential function to the least-square log transformation, since we have an error $\chi_{3}$ that we need to estimate. In previous studies, an a priori constant has to be chosen, and not estimated, for $\chi_{3}$ [10]; then, a fitted least-square log transformation was applied to the cumulative data. In this paper, we estimate the parameters directly using the self-starting functions proposed [40]. In practice, we directly fit the nonlinear exponential cumulative function using *basicTrendline* with functions *Nls* and *SSexp3P* (R-project/basicTrendline). The evaluation of *χ*_1_, *χ*_2_, *χ*_3_ (Table 4) using the cumulative reported symptomatic-and-infectious cases and the direct fit of the exponential curve is summarized in Table 4 and shown in Figure 3 and Figures S1 and S2, in the Supplementary Information (SI) for Qatar.

We verified that the data for Qatar, KSA, Kuwait, New York and Italy fit the model (2) very well on the basis of the results. However, the data for Bahrain, UAE and Oman fit the model appropriately.

The algorithm for determining the initial conditions *S*_0_, *I*_0_ and the parameters τ, ν_1_, ν_2_ on the basis of the reported case data is as follows:

**Step 1**: Since f and ν are fixed, we know that $\nu_{1}=f\nu$ and $\nu_{2}=\left(1-f\right)\nu$.

**Step 2**: Knowing $\chi_{1}$, $\chi_{2}$ and $\chi_{3}$, we calculate the starting point of the pandemic $t_{0}=\frac{1}{\chi_{2}}\left(ln\left(\chi_{3}\right)-ln\left(\chi_{1}\right)\right)$. 

Using $C\left(t\right)=\nu_{1}\overset{t}{\underset{0}{\int }}I\left(h\right)dh$ and $I\left(t\right)=I_{0}exp\left(\chi_{2}(t-t_{o}\right)$), we compute the other parameters using [6,7,8,9,10,11,35,41]:

(3) $$\;\;\;\;\;\;\;\;I_{0}=\frac{\chi_{1}\chi_{2}exp\left(\chi_{2}t_{0}\right)}{f\nu }=\frac{\chi_{3}\chi_{2}}{f\nu }$$

(4) $$\tau =\frac{\chi_{2}+\nu }{S_{0}}\frac{\eta +\chi_{2}}{\nu_{2}+\eta +\chi_{2}}$$

(5) $$U_{0}=\frac{\nu_{2}}{\eta +\chi_{2}}I_{0}=\frac{\left(1-f\right)\nu }{\eta +\chi_{2}}I_{0}$$

**Step 3:**

(6) $$\;\;R_{0}=\frac{\tau S_{0}}{\nu +\mu }\left(1+\frac{\nu_{2}}{\eta }\right)\;\;\;$$

(7) $$=\frac{\chi_{2}+\nu +\mu }{\nu +\mu }\frac{\eta +\chi_{2}}{\left(1-f\right)\left(\nu +\mu \right)+\eta +\chi_{2}}\left(1+\frac{\left(1-f\right)\left(\nu +\mu \right)}{\eta }\right)$$

See Appendix A.1 for more details. Please note that there are two unknown parameters, $U_{0\;}$and $\upsilon_{2}$, which are deduced from cumulative number of reported cases. Using [42], we can show that this problem has a solution, as explained in Appendix A.2. Moreover, on the basis of [43] (Lemma 1) and [44], it is known that this problem has a unique and non-negative solutions.

## 3. Results

The parameters τ,η and ν, and the initial conditions *S*($t_{0}$), *I*($t_{0}$), and *U*($t_{0}$), usually remain unrevealed even for influenza disease outbreaks. Meanwhile, here, we focus on their assessment on the basis of the reported symptomatic-and-infectious cases.

We assume that 1/ν can be between one day and seven days. This is the average time during which infected cases are asymptomatic. In addition, we set 1/η  as between one day and seven days, for the average time during which an infected case is symptomatic. Finally, we identified them, assuming that between 80% and 100% of infectious cases were reported. Accordingly, the *f* value was set between 0.8 and 1. Thus, we fixed *f*, η, ν, α, μ and Λ. Using Equation (7) for the basic reproduction number, we obtained from the data an estimation of the basic reproduction number $R_{0}=2.42$ and an average $R_{0}$ in the GCC, which was 2.20 ± 0.123.

Since *f,* η*,* μ, α and Λ are assumed to be known, we can compute the transmission rate on the basis of Equation (4), as shown in Table 4. For example, for Qatar $\tau =7.1\times 10^{-8}$, $I_{0}=10.1$, $U_{0}=1.3$ and $R_{0}=2.42$. The average transmission rate for GCC was [3.55 $\pm \;3.53]\times 10^{-8}$.

**Figure 4:**
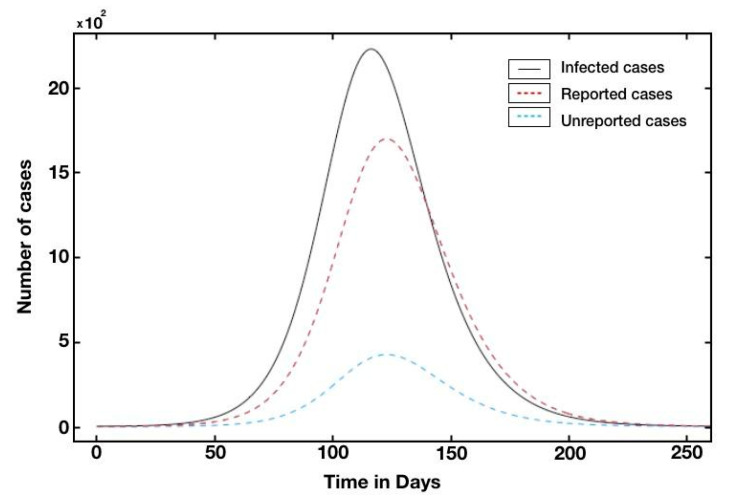
Simulated epidemiological curve without intervention. In this figure, we use *f* = 0.8, *ν* = 1/7, *η* = 1/7, and *S*_0_ = 2.881 × 10^6^. The number of confirmed cumulative cases *t* → *C*(*t*) (black solid line) and *t* → *U*(*t*) is the unreported cases (blue dashed) and (red dashed) of the reported cases in Qatar. We use $\chi_{1}$ = 215.08, $\chi_{2}$ = 0.08, $\chi_{3}$ = 145.48, *t*_0_ = −4.88 and *S*_0_ = 2.881 × 10^6^, which give τ = 7.1 × 10^−8^, *I*_0_ = 10.1, *U*_0_ = 1.3 and *R*_0_ = 2.42.

We also plotted the graphs of *t* → *C*(*t*) (solid black line), *t* → *U*(*t*) (blue dotted), and *t* → *R*(*t*) (red dotted) for Qatar (Figure 4). The turning point was defined as the time at which the red curve reached its maximum value (i.e., the curve of the unaccumulated recorded infectious cases) of between 1500 and 2000 (as shown in Figure 4) and that the turning point was day 109, which was 5 July. The turning point for the UAE was day 100, which corresponds to June 1st, since the UAE was the first country in the Middle East to report a confirmed case of COVID-19 (see Figures S3 and S4 in the Supplementary Information SI).

**Figure 5:**
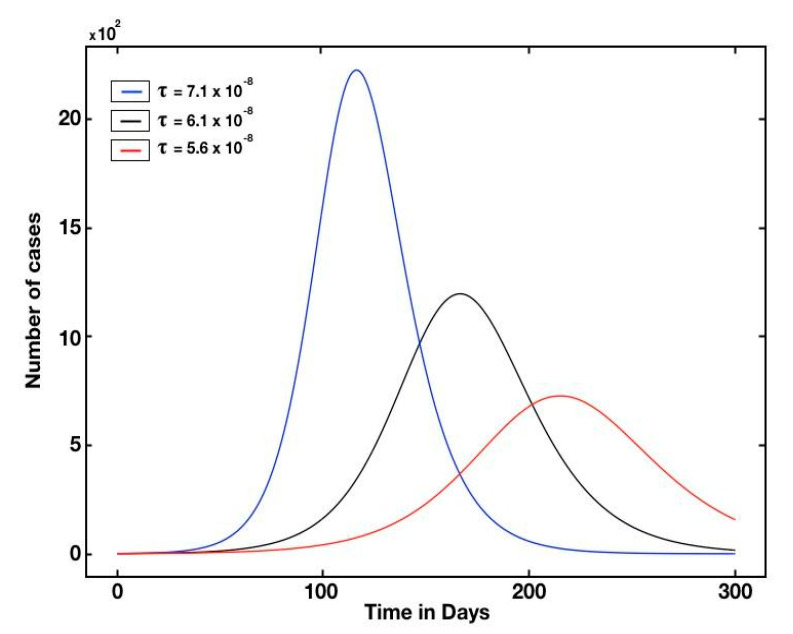
Simulated curves for several transmission rate.

**Figure 6:**
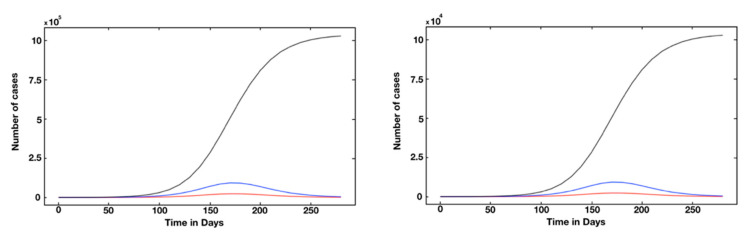
Simulated epidemiological curve without intervention. In this figure, we use *f* = 0.8, *ν* = 1/7, *η* = 1/7, and $S_{0}$ = 2.881 × $10^{6}$. The number of confirmed cumulative cases *t* → *C*(*t*) (black line) and *t* → *U*(*t*) the unreported cases (blue line) and (red line) correspond to the reported cases in Qatar. We use $\chi_{1}$ = 215.08, $\chi_{2}$ = 0.08, $\chi_{3}$ = 145.48, $t_{0}$ = −4.88 and $S_{0}\;$= 2.881 ×$\;10^{6}$. The left side shows the curve without intervention, which gives *τ* = 7.1 × $10^{-8}$. The right side shows the curve with a moderate intervention, which means *τ* = 7.1 × $10^{-8}$ for *t* ∈ [0, 150] and *τ* = 6.1 × $10^{-8}$ for *t* > 150.

Interestingly, although there are different kinetics as per the fitted parameters (Table 5), the fraction of unreported cases was similar among the examined countries/city. This assessment supports the notion that transmission rate reduction is more effective than the disclosure of unreported cases. Subsequently, Figure 5 and Figure 6 show the dynamics of the pandemic with several interventions. For example, the number of reported cases drops by $10^{3}\;$if we use a 14% intervention (with the transmission rate decreasing from 7.1 to 6.1 at the $10^{-8}$ scale) (Figure 5), and that a moderate measure of intervention significantly mitigates the final scale of the epidemic (Figure 6).

*R*_0_ represents the average number of people infected by one infectious individual when there is zero immunity in the population. While we recall the strict isolation steps imposed for all of Qatar and the GCC countries in March 2020 [45], and we take into consideration the number of unstated cases, the corrected estimate was between two and three for Qatar. This assessment is much lower than the reported values. Let us consider $R_{e}$ as the effective reproduction rate resulting from the inclusion of intervention. It is crucial to estimate $R_{e}$ regularly to decide whether to ease off or to make the preventive measures stricter. At the same time, we should account for the acquired immunity within the population and the level of preventative measures being implemented to accurately assess $R_{e}$. Therefore, our proposed approach suggests that the number of unreported cases would enable a better estimate of the effective reproduction rate ($R_{e}$) and a good measure for pandemic control. Thus, we re-evaluated the reproduction numbers while highlighting the existence of unreported cases.

**Figure 7:**
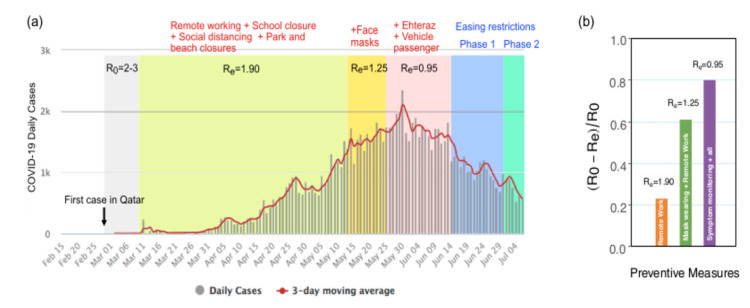
(**a**) A graph summarizing the real infection data with our estimated *R*_0_ and *R_e_* mapped onto phases of intervention measures taken by state of Qatar. (**b**) Relative effectiveness comparisons. The relative effectiveness varies as the effectiveness is quantified as (*R*_0_ − *R*_e_/*R*_0_). The early preventive measures (remote working + school closure + social distancing + park/beach closure) reduced *R_e_* by > 20%. In addition, the additional imposition of nation-wide mask-wearing reduced it further by > 60%. Furthermore, a >81% reduction was attained with the mandatory introduction of the GPS-Bluetooth-based contact-tracing application (Ehteraz) for all the residents on their smartphones.

Figure 7a reports the number of new daily COVID-19 cases reported by Qatar’s authorities during the pandemic between March and July 2020. It shows our estimates of the $R_{e}$ for the state of Qatar. The early preventive measures, such as remote working and schooling, social distancing, and park and beach closure, taken by the state at the beginning of the spread of the virus led to an $R_{e}$ of 1.90. This estimate is lower than the reported $R_{0}$ at the beginning of the pandemic. Starting from May, subsequent additional measures were taken by Qatar, such as an obligation to wear a mask, which resulted in an $R_{e}$ of approximately 1.25. Further reduction of $R_{e}$ may have been achieved by the mandatory contact-tracing mobile application Ehteraz and limiting the number of passengers per vehicle, effective as of 4 June 2020. The interventions implemented beginning in May began to bear fruit, resulting in a peak of infection and an inflection point in early June, when the number of reported cases began to progressively decline. These measures enabled Qatar to start the safe reopening of business in July, and a progressive return to activity throughout the planned phases of reopening while keeping the $R_{e}$ below one. We interpret that this could be achieved by maintaining a constant the level of intervention through mask-wearing and restricted access, in addition to imposing the use of the contact tracing app along with other measures, such massive testing combined with both targeted and random sampling.

For the Qatari population, when investigating the dynamics of non-pharmaceutical intervention such as quarantine, social distancing, mask-wearing, and symptom monitoring (using Ehteraz App, Doha, Qatar), we find that the effectiveness of symptom monitoring, in addition to mask-wearing and quarantine, in controlling the disease is efficient for reducing the effective reproduction number by 81%, while social distancing and quarantine alone reduced the $R_{e}$ by 21%, and mask-wearing with the former intervention accounted for 62% of the reduction (Figure 7b).

For the post-COVID-19 recovery of economies, we propose investing in additional longer-term protection, such as the application of smart anti-viral and anti-bacterial coatings for surfaces that are touched often [46]. This strategy would enable us to provide baseline protection against viral mutation, seasonal flu, and bacterial infections that would greatly help in avoiding overloading healthcare systems. This type of longer-term intervention might play a role in various situations, especially with respect to upcoming major sporting events that the country plans to host, such as the world cup in 2022.

## 4. Conclusions

We applied a modified SIR model with comprehensive consideration of the identification of model parameters using reported case data, both asymptomatic and symptomatic, to model the transmission dynamics of the COVID-19 pandemic for some of the GCC countries and some representative countries in Europe and New York City, taking into consideration the rate of unreported cases and the importance of its estimation in affecting the actual spread of the virus and the level of measures taken to face it. Thus, we estimated the basic reproduction rates $R_{0}\;\mathrm{and}\;R_{e}.$ The objective of the analysis was to identify the early phases of the epidemics, and to predict the next phase and the shape of their evolution, while incorporating unreported cases into the transmission dynamics. The model also aims to highlight the effectiveness of the implementation of major public policies restricting social movement result in a time-dependent exponentially decreasing number of cases to support a safe reopening strategies in the GCC countries. The model can be generalized for a wider range of countries.

Our epidemiological model supports the evaluation of COVID-19 management activities in the early pandemic period, mainly when most of the population is susceptible and the public understanding of the symptoms is degrading. The value of this epidemiological model analysis is its ability to view various aspects, providing the impact of unreported cases. Furthermore, this model illustrates the results of preventive strategies more accurately without a prior knowledge of the consequence of disease incursions. However, there is a limitation in the analysis when using the data sourced from early in the COVID-19 pandemic. Although this study did not take into account the hosts who were immune to COVID-19 either through infection or inoculation, the modified SIR model with a varying number of susceptible hosts at a time can depict the influence in dynamic circumstances.

## Figures and Tables

**Table 1:** The value of *R*_0_ for selected countries.

Countries	*R*_0_	Reference
Iran	2.30	[12]
South Korea	2.60	[13]
Singapore	1.54	[14]
Japan	2.20	[19]
Israel	1.26	[15]
Algeria	2.55	[16]
USA	4.02	[17]
Brazil	2.81	[18]
China	6.6	[20]

**Table 2:** Parameters of the model using the added compartment summarizing the reported and unreported rate.

Symbol	Interpretation
$t_{0}$	Time at which the epidemic started
$S_{0}$	Number of susceptible at time
$I_{0}$	Number of asymptomatic infectious at time
$U_{0}$	Number of unreported symptomatic infectious at time
*1/* ν	Average time during which asymptomatic are asymptomatic
*f*	Fraction of asymptomatic that become reported symptomatic
$\nu_{1}=f\nu$	Rate at which asymptomatic become reported symptomatic
μ	Natural death rate
Λ	Number of births per unit time
α	Fraction of infectives recovering with immunity against reinfection
$\nu_{2}=\nu -\nu 1$	Rate at which asymptomatic become unreported symptomatic
1/η	Average time symptomatic infectious have symptoms

**Table 3:** Cumulative reported case data between the 2nd and 8th of May 2020, reported for Qatar by Worldometers ^a^.

Cumulative	2nd	3rd	4rd	5th	6th	7th	8th
Reported cases	14,872	15,551	16,191	17,142	17,972	18,020	18,321
Predicted cases	15,138	16,524	17,027	19,658	19,427	19,701	19,890

^a^ Worldometers manually analyzes, validates, and aggregates data from thousands of sources in real time and provides global COVID-19 live statistics for a wide audience of caring people around the world. Data is also trusted and used by the UK Government, Johns Hopkins CSSE, The Financial Times, The New York Times, Business Insider, BBC, and many others.

**Table 4:** Estimation of the parameters *χ*_1_, *χ*_2_, *χ*_3_, *t*_0_ and *τ for Qatar* using the cumulative reported cases. Λ is per 1000 people.

$\chi_{1}$	$\chi_{2}$	$\chi_{3}$	$t_{0}$	τ	Λ	µ
215.08	0.08	145.48	−4.88	7.1 × 10^−8^	9.40	1.20%

**Table 5:** Summary table of *t*_0_, *τ*, *I*_0_, *U*_0_ and *R*_0_ for several countries. For example, *t*_0_ for Qatar is −5, which means that the estimated starting day of the pandemic is 5 days earlier than the stated day, which means that the estimated starting date of the pandemic is the 24 February 2020. The number of asymptomatic infectious at time *t*_0_ is *I*_0_ = 10.1, and the number of unreported symptomatic infectious at time *t*_0_ is *U*_0_ = 1.3. New York city was used for purposes of comparison, since it was the center of the American COVID-19 outbreak.

Country	*t*_0_	*τ*	*I*_0_	*U*_0_	*R*_0_
Qatar	−5	7.10 × 10^−8^	10.1	1.3	2.42
Saudi Arabia	−1	0.58 × 10^−8^	26.4	3.3	2.45
UAE	−1	7.60 × 10^−8^	12.4	1.8	2.19
Bahrain	−20	10.4 × 10^−8^	09.8	1.5	2.19
Kuwait	−22	3.74 × 10^−8^	11.0	1.4	2.37
Oman	−7	3.39 × 10^−8^	14.9	2.2	2.20
France	−6	0.38 × 10^−8^	09.2	0.9	2.84
Italy	−7	0.42 × 10^−8^	17.1	7.3	2.80
New York	−4	6.48 × 10^−8^	03.1	1.0	3.62

## Data Availability

The raw/processed data can be obtained by contacting the authors.

## Supplementary Materials

The following are available online at https://www.mdpi.com/article/10.3390/biology10060463/s1, Figure S1: SIR Model (black line) vs real cumulative reported values (blue dotted) for GCC. Top left: Bahrain, top right: Oman, bottom left: Kuwait and bottom right: KSA. Figure S2: SIR Model (black line) vs real cumulative reported values (blue dotted) for non GCC (Spain on top left, New York City on top right, France on bottom left and Italy on bottom right). Figure S3: Simulated reported versus unreported for KSA (top left), Oman (top right), Bahrain (bottom left) and Kuwait (bottom right). Figure S4: Simulated reported versus unreported for Spain (top left), France (top right), Italy (bottom left) and New York city (bottom right).

## Appendix A

### Appendix A.1

Derivation of *U*_0_ and *I*_0_:

Using (2) we have ν

$$U\left(t\right)=\frac{1}{\tau S_{0}}(I^{\prime }\left(t\right)+\left(\nu +\mu \right)I\left(t\right))-I\left(t\right)$$

On the other hand, since we assume that *C*(*t*) has an exponential behavior *C*(*t*) = $\chi_{1}\exp\;\left(\chi_{2}t\right)+\chi_{3}$

(A1) $$\{\begin{array}{c} U\left(t\right)=\frac{1}{\tau S_{0}}\left(I^{\prime }\left(t\right)+\left(\nu +\mu \right)I\left(t\right)\right)-I\left(t\right)\\ I\left(t\right)=I_{0}\exp\left(\chi_{2}\left(t-t_{0}\right)\right)\\ t_{0}=\frac{1}{\chi_{2}}\left(\log\left(\chi_{3}\right)-\log\left(\chi_{1}\right)\right) \end{array}$$

We have also

(A2) $$\{\begin{array}{c} I^{\prime }\left(t\right)=\tau_{0}S_{0}\left(I\left(t\right)+U\left(t\right)\right)-\left(\nu +\mu \right)I\left(t\right)\\ U^{\prime }\left(t\right)=\nu_{2}I\left(t\right)-\eta U\left(t\right) \end{array}$$

Using (A1) we have

$$\{\begin{array}{c} I^{\prime }\left(t\right)=I_{0}\chi_{2}\exp\left(\chi_{2}\left(t-t_{0}\right)\right)\\ U^{\prime }\left(t\right)=U_{0}exp(\chi_{2}\left(t-t_{0}\right)) \end{array}$$

and

$$\;\;\;I^{\prime }\left(t\right)=\chi_{2}I_{0}\exp\left(\chi_{2}\left(t-t_{0}\right)\right)$$

$$\;\;U^{’}\left(t\right)=\chi_{2}I_{0}\exp(\chi_{2}\left(t-t_{0}\right)$$

Using (2) and (A2), we have

(A3) $$\;\{\begin{array}{c} \chi_{2}I_{0}=\tau S_{0}\left(I_{0}+U_{0}\right)-\left(\nu +\mu \right)I_{0}\\ \chi_{2}U_{0}=\nu_{2}I_{0}-\eta U_{0} \end{array}$$

Using Liu et al. [6] and [7], $\chi_{2}\;$is the dominant eigenvalue of (A2) and $(I_{0},U_{0})\;$is a positive eigenvector associated with $\chi_{2}$.

If we divide (A3) by $I_{0}\;$we have:

$$\{\begin{array}{c} \chi_{2}=\tau S_{0}\left(1+U_{0}\right)-\left(\nu +\mu \right)\\ ⬚\\ \frac{U_{0}}{I_{0}}=\frac{\nu_{2}}{\eta +\chi_{2}} \end{array}$$

### Appendix A.2

Here, we adapt the same procedure derived in the work of Magal and Webb (2017) [42] to prove that we can find a solution to the unknown $\tau ,\;\upsilon_{1\;}$and $\upsilon_{2\;}$ under one assumption, which is $\frac{C\left(t_{p}\right)}{C_{\infty }}<\frac{1}{2}$.

In fact, from the SIR model Equation (2), we have:

$$S\left(t\right)+I\left(t\right)+\left(\nu_{1}+\nu_{2}+\mu \right)\int_{0}^{t}I\left(h\right)dh=S_{0}+I_{0}$$

and

$$S\left(t\right)=S_{0}\exp(-\tau \int_{0}^{t}I\left(h\right)dh)$$

Therefore

$$\underset{t\to \infty }{\lim}S\left(t\right)=S_{0}>0\;\mathrm{and}\;\underset{t\to \infty }{\lim}I\left(t\right)=I_{0}=0$$

and

$$S_{\infty }=S_{0}+I_{0}+\frac{\nu_{1}+\nu_{2}+\mu }{\tau }\;\log\;\frac{S_{\infty }}{S_{0}}$$

$$C\left(t\right)=\nu \int_{0}^{t}I\left(h\right)dh=\frac{\nu_{1}}{\tau }\log\frac{S_{0}}{S_{t}}\;\mathrm{and}\;C_{\infty }=\underset{t\to \infty }{\lim}C\left(t\right)=\frac{\nu_{1}}{\tau }\log\frac{S_{0}}{S_{\infty }}$$

which implies that:

$$S_{0}\exp(-\frac{\tau }{\upsilon_{1}}C_{\infty })=S_{0}+I_{0}-\frac{\nu_{1}+\nu_{2}+\mu }{\nu_{1}}C_{\infty }$$

Skipping some details and using the paper by Magal and Webb, we can derive the following three equations:

(A4) $$S_{0}\exp\left(-\frac{\tau }{\nu_{1}}C_{\infty }\right)=(S_{0}+I_{0})-\frac{\nu_{1}+\nu_{2}+\mu }{\nu_{1}}C_{\infty }$$

(A5) $$C\left(t_{p}\right)=\frac{\nu_{1}}{\tau }log\frac{\tau S_{0}}{\nu_{1}+\nu_{2}+\mu }$$

or equivalently

$$\frac{\nu_{1}+\nu_{2}+\mu }{\tau }=S_{0}\exp(-\frac{\tau }{\nu_{1}}C\left(t_{p}\right))$$

(A6) $$\left(S_{0}+I_{0})-I\left(t_{p}\right)=\frac{\nu_{1}+\nu_{2}+\mu }{\tau }(1-\log\frac{\nu_{1}+\nu_{2}+\mu }{\tau S_{0}}\right)$$

(A5) implies that

$$\nu_{1}+\nu_{2}+\mu =\tau S_{0}\exp\left(-\frac{\tau }{\nu_{1}}C\left(t_{p}\right)\right)$$

If we substitute that in (A4), we have

$$S_{0}\exp\left(-\frac{\tau }{\nu_{1}}C_{\infty }\right)=\left(S_{0}+I_{0}\right)-\left(\frac{\tau S_{0}}{\nu_{1}}\exp(-\frac{\tau }{\nu_{1}}C\left(t_{p}\right)\right)C_{\infty }$$

and we have

$$\left(S_{0}+I_{0}\right)=S_{0}\exp(-\frac{\tau }{\nu_{1}}C_{\infty })+\left(\frac{\tau S_{0}}{\nu_{1}}\exp(-\frac{\tau }{\nu_{1}}C\left(t_{p}\right)\right)C_{\infty }$$

If we divide the last equation by $S_{0}$, we have

$$\exp(-\frac{\tau }{\nu_{1}}C_{\infty })+\left(\frac{\tau }{\nu_{1}}\exp(-\frac{\tau }{\nu_{1}}C\left(t_{p}\right)\right)C_{\infty }-1=\frac{I_{0}}{S_{0}}$$

We can write this as a function in the form

$$g\left(x\right)=\frac{I_{0}}{S_{0}}=\exp\left(-\alpha x\right)+\alpha x\exp\left(-\alpha \beta x\right)-1$$

with

$$x=\frac{\tau }{\nu_{1}},\;\alpha =C_{\infty },\;\;\beta =\frac{C\left(t_{p}\right)}{C_{\infty }}$$

Using proposition (3.1) from the paper by Magal and Webb [42], this function has two positive solutions, and if one of the solutions $x^{*}=\frac{\tau }{\nu_{1}}$, then using (A5) and (A6), we can calculate $\tau ,\;\nu_{1}$ and $\nu_{2}.\;$More details are given in Ref. [42].
