# Supplementary material for: Using Unstated Cases to Correct for COVID-19 Pandemic Outbreak and Its Impact on Easing the Intervention for Qatar
Source: Biology (Basel). 2021 May 24;10(6):463. doi: 10.3390/biology10060463 (PMC8225146; doi:10.3390/biology10060463)
Supplement: Supplementary file 1 [file biology-10-00463-s001.zip › biology-1160155-supplementary.pdf]

biology

MDPI

Article

Using Unstated Cases to Correct for COVID-19 Pandemic Outbreak and Its Impact on Easing the Intervention for Qatar.

Narjiss Sallahi [1], Heesoo Park [2], Fedwa El Mellouhi [2], Mustapha Rachdi [3], Idir Ouassou [4], Samir Belhaouari [5], Abdelilah Arredouani [6] and Halima Bensmail [7,*]

1 National Institute of Posts and Telecommunications (INPT), Rabat, Morocco; narjiss.sallahi@gmail.com
2 Qatar Environment and Energy Research Institute, Hamad Bin Khalifa University, P.O. BOX 34110, Doha, Qatar; {hpark,felmellouhi}@hbku.edu.qa
3 University of Grenoble, Data sciences Project, Grenoble, France; mustapha.rachdi@univ-grenoble-alpes.fr
4 University Qaddi Ayyad, Marrakech, Morocco; i.ouassou@uca.ac.ma
5 ICT Department, Hamad Bin Khalifa University; sbelhaouari@hbku.edu.qa
6 Qatar Biomedical Research Institute, Hamad Bin Khalifa University; aarredouani@hbku.edu.qa
7 Qatar Computing Research Institute, Hamad Bin Khalifa University; hbensmail@hbku.edu.qa
* Correspondence: hbensmail@hbku.edu.qa; Tel.:974 5527 8824

Citation: Sallahi, N.; Park, H.; Mellouhi, F.E.; Rachdi, M.; Ouassou, I.; Belhaouari, S.; Arredouani, A.; Bensmail, H. Using Unstated Cases to Correct for COVID-19 Pandemic Outbreak and Its Impact on Easing the Intervention for Qatar. *Biology* **2021**, *10*, 463. https://doi.org/10.3390/biology10060463

Academic Editor: Jukka Finne

Received: 7 April 2021
Accepted: 19 May 2021
Published: 24 May 2021

Publisher's Note: MDPI stays neutral with regard to jurisdictional claims in published maps and institutional affiliations.

Copyright: © 2021 by the authors. Licensee MDPI, Basel, Switzerland. This article is an open access article distributed under the terms and conditions of the Creative Commons Attribution (CC BY) license (http://creativecommons.org/licenses/by/4.0/).

Supplementary file:

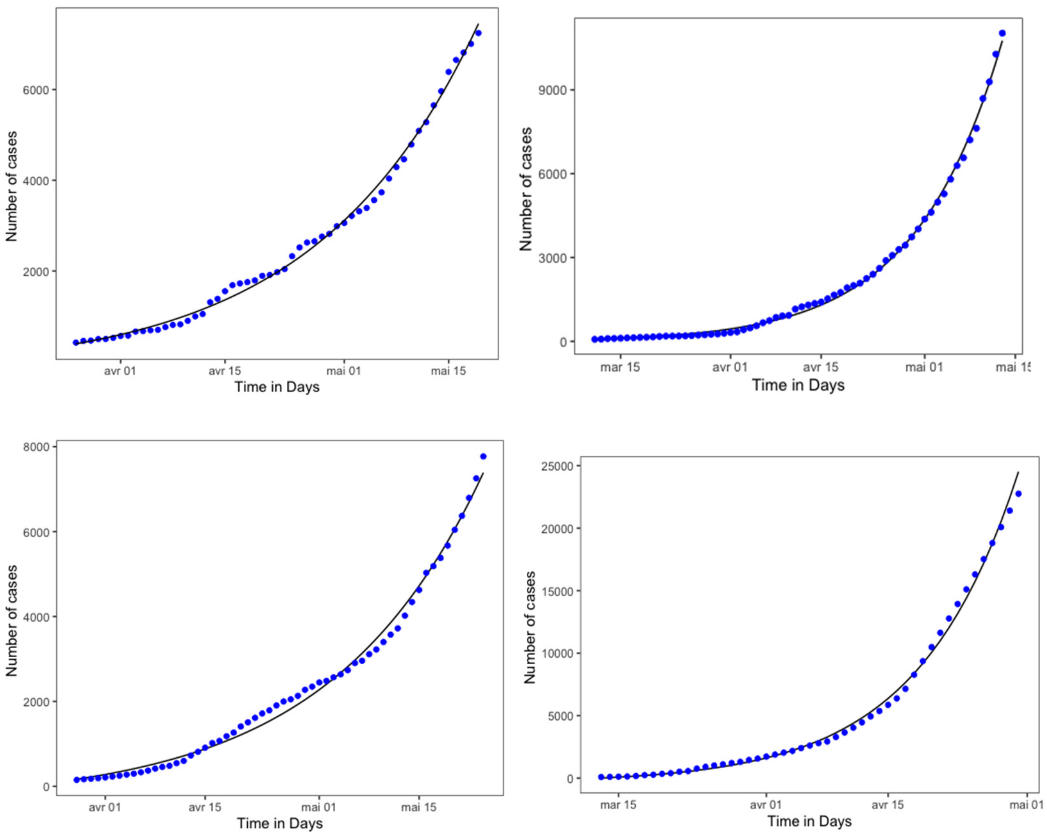

Figure S1: SIR Model (black line) vs real cumulative reported values (blue dotted) for GCC. Top left: Bahrain, top right: Oman, bottom left: Kuwait and bottom right: KSA.

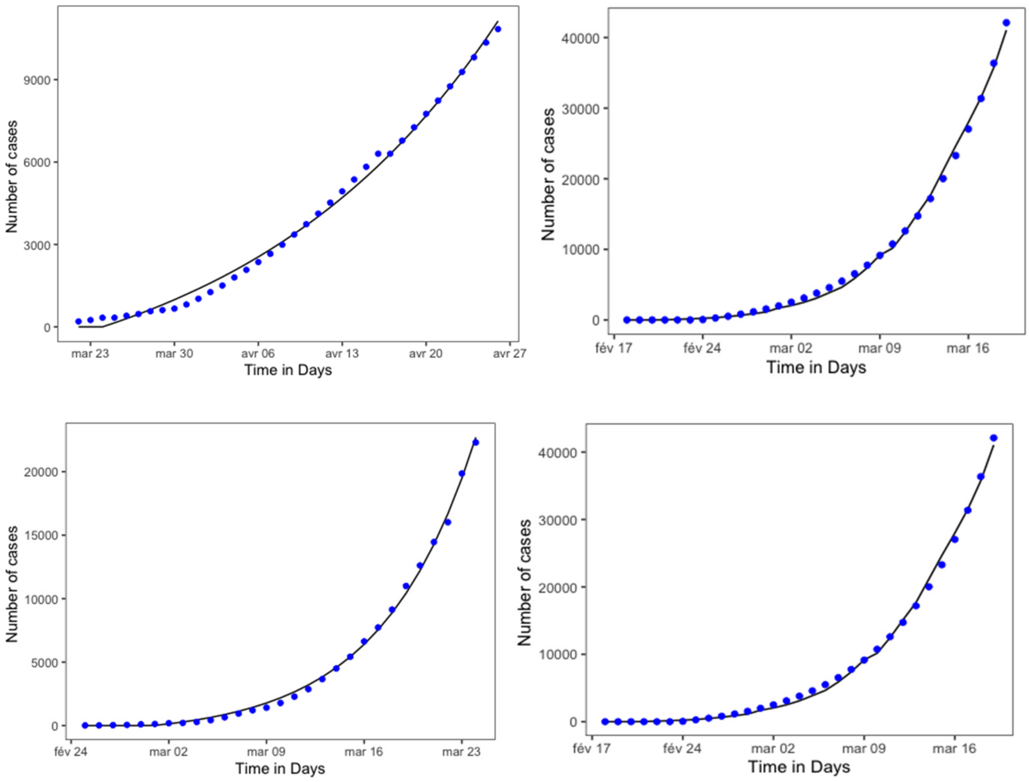

Figure S2: SIR Model (black line) vs real cumulative reported values (blue dotted) for non GCC (Spain on top left, New York City on top right, France on bottom left and Italy on bottom right).

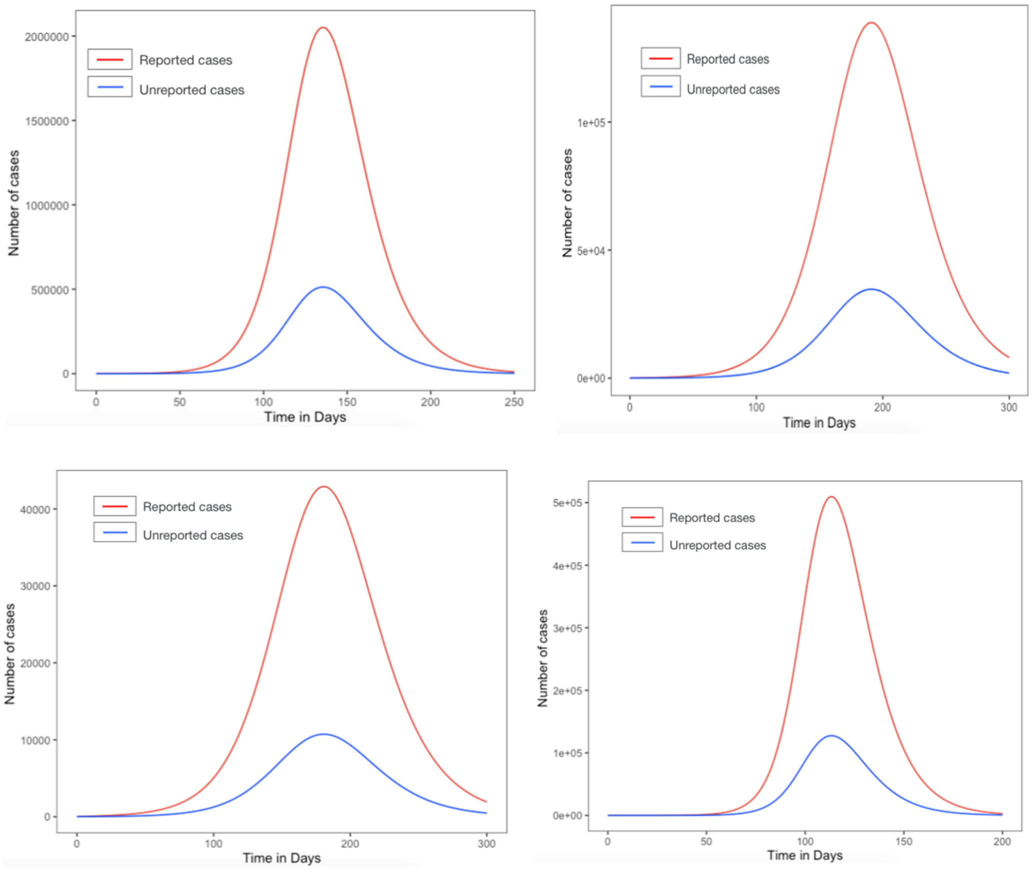

Figure S3: Simulated reported versus unreported for KSA (top left), Oman (top right), Bahrain (bottom left) and Kuwait (bottom right).

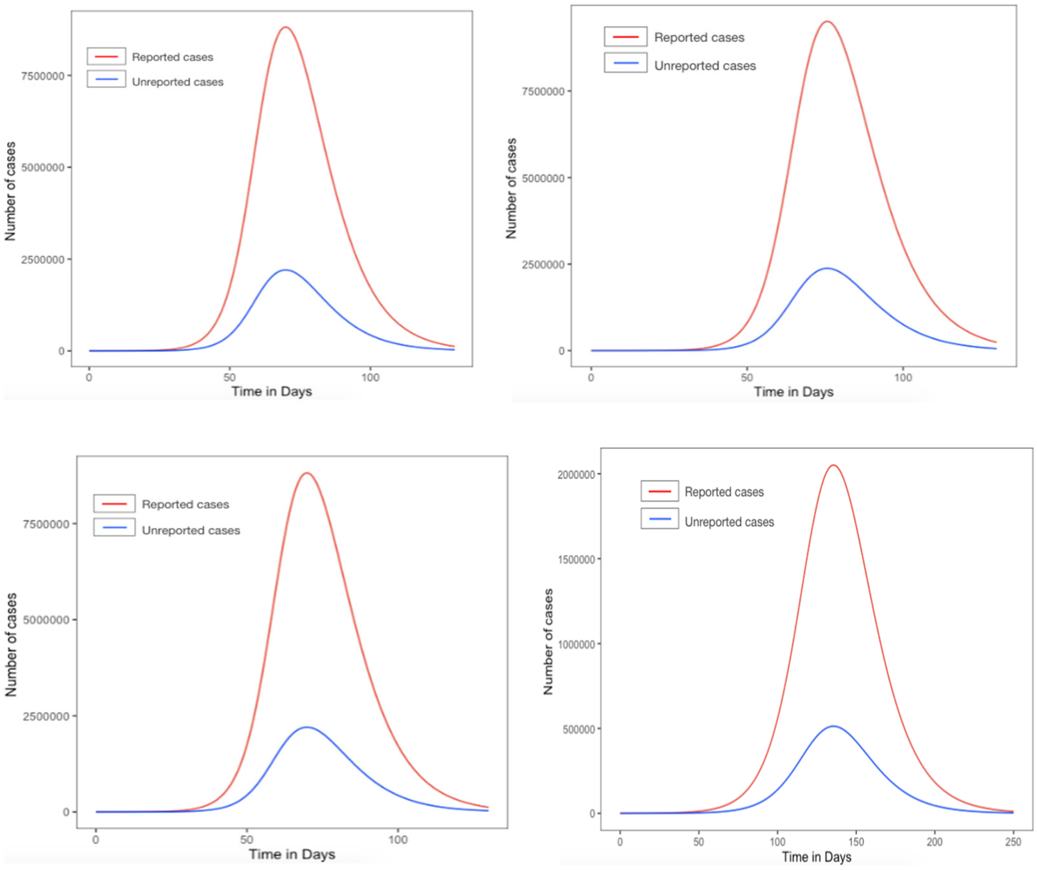

Figure S4: Simulated reported versus unreported for Spain (top left), France (top right), Italy (bottom left) and New York city (bottom right).
